# Determining arterial wave transit time from a single aortic pressure pulse in rats: vascular impulse response analysis

**DOI:** 10.1038/srep40998

**Published:** 2017-01-19

**Authors:** Ru-Wen Chang, Chun-Yi Chang, Liang-Chuan Lai, Ming-Shiou Wu, Tai-Horng Young, Yih-Sharng Chen, Chih-Hsien Wang, Kuo-Chu Chang

**Affiliations:** 1 Department of Physiology, College of Medicine, National Taiwan University, Taipei, 100, Taiwan; 2 Department of Emergency Medicine, National Taiwan University Hospital, Chu-Tung Branch, Hsin-Chu, 310, Taiwan; 3 Department of Internal Medicine, National Taiwan University Hospital, Taipei, 100, Taiwan; 4 Institute of Biomedical Engineering, College of Medicine and Engineering, National Taiwan University, Taipei, 100, Taiwan; 5 Department of Surgery, National Taiwan University Hospital, Taipei, 100, Taiwan; 6 Department of Surgery, National Taiwan University Hospital, Hsin-Chu Branch, Hsin-Chu, 300, Taiwan

## Abstract

Arterial wave transit time (*τ*_*w*_) in the lower body circulation is an effective biomarker of cardiovascular risk that substantially affects systolic workload imposed on the heart. This study evaluated a method for determining *τ*_*w*_ from the vascular impulse response on the basis of the measured aortic pressure and an assumed triangular flow (*Q*^tri^). The base of the unknown *Q*^tri^ was constructed with a duration set equal to ejection time. The timing of the peak triangle was derived using a fourth-order derivative of the pressure waveform. Values of *τ*_*w*_s obtained using *Q*^tri^ were compared with those obtained from the measure aortic flow wave (*Q*^m^). Healthy rats (*n* = 27), rats with chronic kidney disease (CKD; *n* = 22), and rats with type 1 (*n* = 22) or type 2 (*n* = 11) diabetes were analyzed. The cardiovascular conditions in the CKD rats and both diabetic groups were characterized by a decrease in *τ*_*w*_s. The following significant relation was observed (*P* < 0.0001): *τ*_*w*_^triQ^ = −1.5709 + 1.0604 × *τ*_*w*_^mQ^ (*r*^2^ = 0.9641). Our finding indicates that aortic impulse response can be an effective method for the estimation of arterial *τ*_*w*_ by using a single pressure recording together with the assumed *Q*^tri^.

The systolic workload imposed on the heart is substantially impaired by increased arterial stiffness and excessive pressure pulsatility, which have emerged as significant predictors of cardiovascular risk in diseased populations^1^,^2^. Progressive arterial stiffening, together with changes in aortic geometry, local arterial branching, and lumen narrowing, creates a mismatch in vascular impedance^3^,^4^. This impedance mismatch causes partial reflections of the forward pressure wave (i.e., a reflected wave traveling back to the central aorta), impairing the systolic loading condition for the left ventricle coupled to the arterial system^5^,^6^. Thus, vascular reflections caused by arterial stiffening affect the pressure wave shape in the proximal aorta^7^, and the severity of their effects depends on the magnitude and time of return from the peripheral circulation.

A complete description of arterial wave properties, including wave transit time (*τ*_*w*_) and wave reflection magnitude (*RM*) or wave reflection index (*RI*), requires simultaneous records of aortic pressure and flow signals. In clinical practice, it is helpful to measure arterial wave properties by using minimally invasive physiological signals if possible. Analysis of the aortic pressure waveform is a method frequently used to assess large artery properties and arterial stiffness. In 2006, Westerhof *et al*.^8^ provided a novel method for calculating pressure wave reflection on the basis of the measured aortic pressure alone. Using an unknown aortic flow with a triangular shape, they successfully separated the measured pressure pulse into its forward and backward components to calculate the *RM* and *RI*. However, the return time of the reflected wave from peripheral circulation was not quantified in their study.

The return time of the reflected wave is the time difference between the forward and backward pressure waves. In 2008, Qasem and Avolio^9^ estimated pulse transit time from waveform decomposition of central aortic pressure by using a cross-correlation of modified forward and backward waves. Another method for determining wave transmission time entails using the vascular impulse response function in the arterial system. In 1980, Sipkema *et al*.^10^ revealed the presence of clear peaks in impulse response, and the measurement of the time intervals between these peaks enabled them to calculate the return time of the reflected wave traveling back to the ascending aorta.

In this study, we evaluated a method for determining *τ*_*w*_ from the impulse response of the filtered aortic input impedance (*Z*_*i*_) on the basis of the measurement of a single aortic pressure pulse and an assumed triangular flow wave (*Q*^tri^). The base of the unknown *Q*^tri^ was constructed with a duration set equal to ejection time. The timing of the peak triangle, which was close to the peak of the measured aortic flow (*Q*^m^), was derived from the fourth-order derivative of the pressure waveform. *RM* and *RI* were calculated from the forward and backward components of the pressure pulse by using wave separation analysis. Healthy rats (NC), rats with chronic kidney disease (CKD), and rats with type 1 or type 2 diabetes mellitus (DM) were analyzed. The values of *τ*_*w*_ and *RM* and *RI* obtained using *Q*^tri^ were compared with those obtained from *Q*^m^.

## Results

Measurements were performed in the NC, CKD, and type 1 and type 2 DM groups. Their baseline characteristics are displayed in Table 1. The CKD rats exhibited impaired renal function, as manifested by increased serum creatinine (SCr) and blood urea nitrogen (BUN). The hemodynamic condition in the rats with CKD was characterized by (1) no alteration in basal heart rate (*HR*) and cardiac output (*CO*) and (2) an augmentation in systolic (*P*_*s*_), diastolic (*P*_*d*_), and mean (*P*_*m*_) pressures in the aorta. The notable increase in *P*_*m*_ was the dominant factor responsible for the increase of 52.7% in total peripheral resistance (*R*_*p*_) in the CKD rats compared with the NC. The rats with streptozotocin (STZ)-induced type 1 DM had higher blood glucose levels associated with a decrease in body weight (BW). Table 1 also illustrates that, partially protected by nicotinamide (NA), the STZ-NA-induced type 2 DM elicited moderate and stable hyperglycemia and prevented STZ-induced hypoinsulinemia and BW loss. Both diabetic groups showed significant declines in *HR*, but not in aortic pressure profiles. With unchanged *P*_*m*_, the decreased *CO* was the predominant factor responsible for an increase in *R*_*p*_ in the rats with type 2 DM.

**Figure 1:**
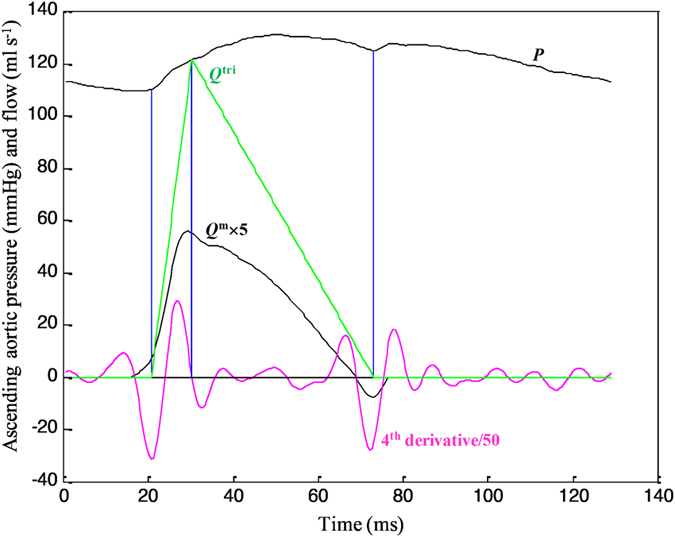
Construction of an uncalibrated triangular flow from the measured pressure waveform in a healthy rat. The start and end time points of the base of the triangle are identified as the intersection of two tangential lines around the foot of the pressure wave (first vertical blue line) and that around the incisura (third vertical blue line), respectively. After ejection commenced, the peak of the triangle is set at the first zero crossing (from above to below) on the fourth-order derivative of the aortic pressure wave (second vertical blue line). Thus, the uncalibrated flow is approximated by a triangle, which is represented by the green line. *P*, measured aortic pressure; *Q*^m^, measured aortic flow; *Q*^tri^, uncalibrated triangular flow.

Figure 1 exemplifies the construction of an uncalibrated triangular flow from the measured pressure waveform in a healthy rat. The base of the triangle is constructed with a duration set equal to ejection time. The start and end time points of ejection are identified as the intersection of two tangential lines around the foot of the pressure wave (first vertical blue line) and that around the incisura (third vertical blue line), respectively. After ejection commenced, the peak of the triangle is set at the first zero crossing (from above to below) on the fourth-order derivative of the aortic pressure wave (second vertical blue line). Thus, the uncalibrated flow is approximated by a triangle, which is represented by the green line.

**Figure 2:**
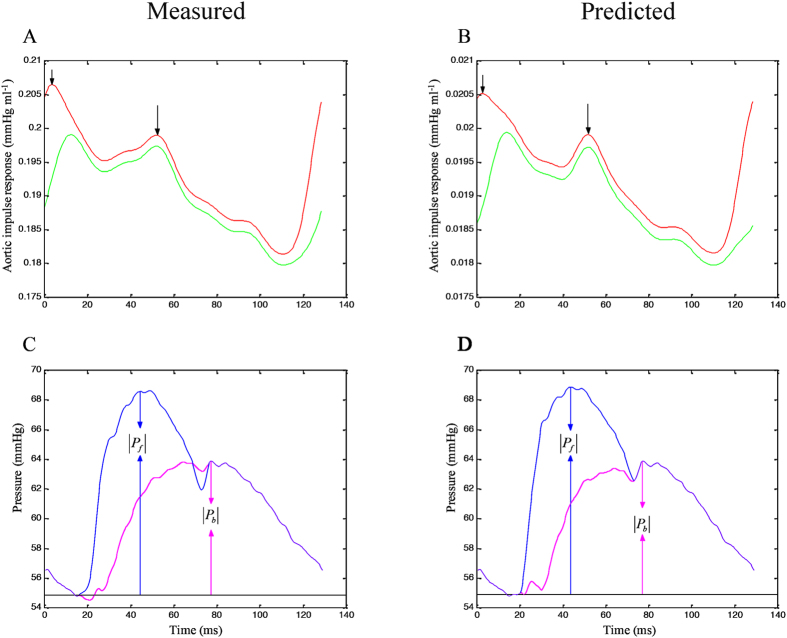
Similarity between the measured aortic impulse response obtained from the measured pressure and *Q*^m^ (**A**) and the predicted aortic impulse response obtained from the measured pressure and *Q*^tri^ (**B**). In (**A** and **B**), the green line indicates that the *Z*_*c*_ component of the vascular impulse response has been removed. One-half of the time difference between the appearance of the reflected peak (long arrow) and the initial peak (short arrow) approximates *τ*_*w*_ in the lower body circulation. The similarities between the measured forward and backward pressure waves (**C**) and the predicted forward and backward pressure waves (**D**) are also presented. In (**C** and **D**), the amplitudes (peak–trough) of the backward and forward pressure waves are represented by |*P*_*b*_| and |*P*_*f*_|, respectively. *Q*^m^, measured aortic flow; *Q*^tri^, uncalibrated triangular flow; *Z*_*c*_, aortic characteristic impedance; *τ*_*w*_, wave transit time.

Figure 2 shows the similarity between the measured aortic impulse response obtained from the measured pressure and *Q*^m^ (A) and the predicted aortic impulse response obtained from the measured pressure and *Q*^tri^ (B). In A and B, the dashed green line indicates that the aortic characteristic impedance (*Z*_*c*_) component of the vascular impulse response has been removed. One-half of the time difference between the appearance of the reflected peak (long arrow) and the initial peak (short arrow) approximates arterial *τ*_*w*_ in the lower body circulation. The similarities between the measured forward and backward pressure waves (C) and the predicted forward and backward pressure waves (D) are also presented. In C and D, the amplitudes (peak–trough) of the backward and forward pressure waves are represented by |*P*_*b*_| and |*P*_*f*_|, respectively.

**Figure 3:**
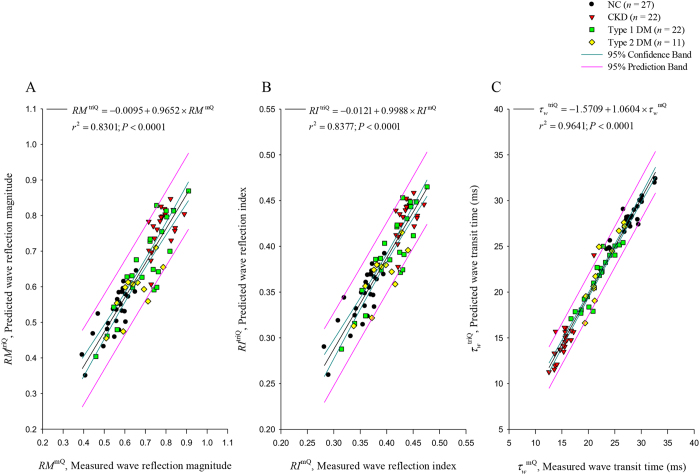
Relations between *RM* (**A**), *RI* (**B**), and *τ*_*w*_ (**C**) calculated from the measured pressure and *Q*^m^ (*RM*^mQ^, *RI*^mQ^, and *τ*_*w*_^mQ^; on the horizontal axes) and *RM, RI*, and *τ*_*w*_ calculated from the measured pressure and assumed *Q*^tri^ (*RM*^triQ^, *RI*^triQ^, and *τ*_*w*_^triQ^; on the vertical axes). *Q*^m^, measured aortic flow; *Q*^tri^, uncalibrated triangular flow; *RM*, wave reflection magnitude; *RI*, wave reflection index; *τ*_*w*_, arterial wave transit time in the lower body circulation; NC, normal controls; CKD, rats with chronic kidney disease; type 1 DM, streptozotocin-induced diabetic rats; type 2 DM, streptozotocin–nicotinamide-induced diabetic rats.

Figure 3 displays relations between *RM, RI*, and *τ*_*w*_ calculated from the measured pressure and *Q*^m^ (*RM*^mQ^, *RI*^mQ^, and *τ*_*w*_^mQ^; on the horizontal axes) and *RM, RI*, and *τ*_*w*_ calculated from the measured pressure and *Q*^tri^ (*RM*^triQ^, *RI*^triQ^, and *τ*_*w*_^triQ^; on the vertical axes). Figure 3A shows a significant regression line for *RM: RM*^triQ^ = −0.0095 + 0.9652 × *RM*^mQ^ (*r*^2^ = 0.8301; *P* < 0.0001). The regression equation of *RI*^triQ^ = −000121 + 0.9988 × *RI*^mQ^ (*r*^2^ = 0.8377; *P* < 0.0001) is provided in Fig. 3B. Figure 3C displays the regression line between *τ*_*w*_^triQ^ and *τ*_*w*_^mQ^ as being *τ*_*w*_^triQ^ = −1.5709 + 1.0604 × *τ*_*w*_^mQ^ (*r*^2^ = 0.9641; *P* < 0.0001).

**Figure 4:**
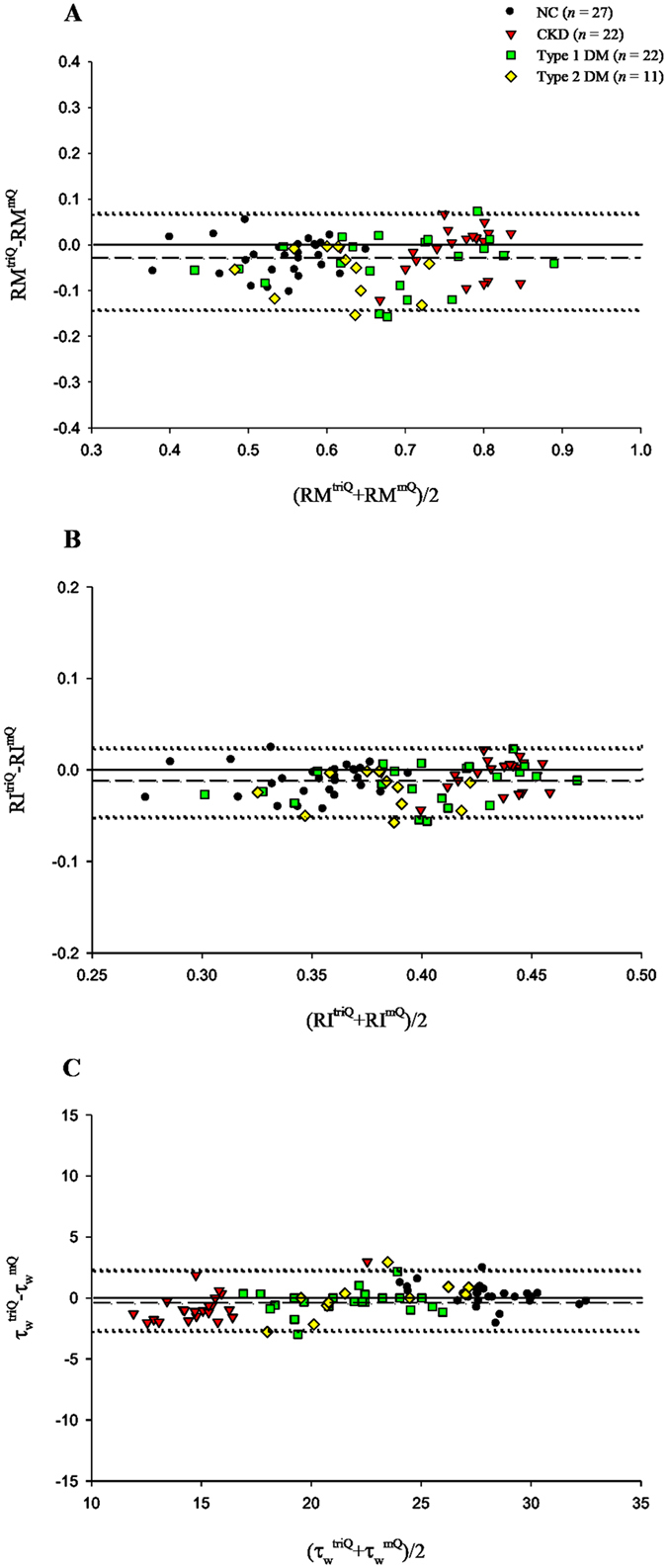
Bland–Altman plots of *RM* (**A**), *RI* (**B**), and *τ*_*w*_ (**C**). Dashed lines represent averages; dotted lines denote 95% confidence intervals. *τ*_*w*_, arterial wave transit time in the lower body circulation; *RM*, wave reflection magnitude; *RI*, wave reflection index; NC, normal controls; CKD, rats with chronic kidney disease; type 1 DM, streptozotocin-induced diabetic rats; type 2 DM, streptozotocin–nicotinamide-induced diabetic rats.

Figure 4 presents a Bland–Altman plot for *RM* (A), *RI* (B), and *τ*_*w*_ (C), with a mean difference of −0.0328, −0.0126, and −0.2318 ms, respectively.

**Figure 5:**
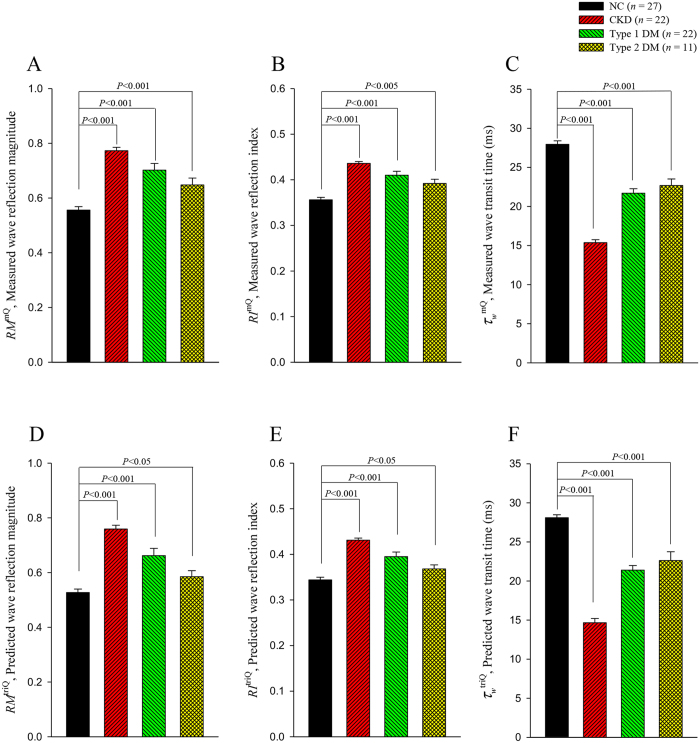
Effects of chronic kidney disease and type 1 and type 2 diabetes on *RM*^mQ^ (**A**), *RI*^mQ^ (**B**), and *τ*_*w*_^mQ^ (**C**), calculated from the measured pressure and *Q*^m^, and their effects on *RM*^triQ^ (**D**), *RI*^triQ^ (**E**), and *τ*_*w*_^triQ^ (**F**), calculated from the measured pressure and assumed *Q*^*tri*^. *Q*^m^, measured aortic flow; *Q*^tri^, uncalibrated triangular flow; *RM*, wave reflection magnitude; *RI*, wave reflection index; *τ*_*w*_, arterial wave transit time in the lower body circulation; NC, normal controls; CKD, rats with chronic kidney disease; type 1 DM, streptozotocin-induced diabetic rats; type 2 DM, streptozotocin–nicotinamide-induced diabetic rats.

Figure 5 shows the effects of the experimental CKD and type 1 and type 2 DM on the arterial wave properties derived from the measured pressure and *Q*^m^, including *RM*^mQ^ (5 A), *RI*^mQ^ (5B), and *τ*_*w*_^mQ^ (5 C). In comparison with the NC, the CKD rats exhibited a higher magnitude of pulse wave reflection, expressed in the form of *RM*^mQ^ and *RI*^mQ^. The CKD rats also exhibited shortened *τ*_*w*_^mQ^. In addition, both diabetic groups exhibited a significant increase in arterial *RM*^mQ^ and *RI*^mQ^, with a diabetes-associated reduction in arterial *τ*_*w*_^mQ^. The parameters calculated using *Q*^tri^ for describing the effects of CKD and DM on arterial wave properties are provided in Fig. 5D (*RM*^triQ^), 5E (*RI*^triQ^), and 5 F (*τ*_*w*_^triQ^). The CKD rats and both diabetic groups exhibited a deterioration in *RM*^triQ^, *RI*^triQ^, and *τ*_*w*_^triQ^, which showed similar statistical significance to that of their measured counterparts (i.e., *RM*^mQ^, *RI*^mQ^, and *τ*_*w*_^mQ^).

## Discussion

In 2006, Westerhof *et al*.^8^ demonstrated an accurate estimate of *RM* and *RI* obtained from the measurement of a single pressure pulse and an uncalibrated triangular flow pulse for ventricular ejection. The present study elaborates this concept by determining *τ*_*w*_ by using an aortic impulse response analysis. We discovered that in addition to wave reflection magnitude measures, the arterial *τ*_*w*_ in the lower body circulation could be approximately estimated using the measured aortic pressure alone in various diseased animals.

It is generally recognized that *Z*_*i*_ can completely describe the physical properties of an arterial system^4^,^11^. Another method of characterizing arterial systems entails using the aortic impulse response function. Conceptually, the impulse response of any linear, time−invariant system, which the vascular system approximates^4^, may be considered as output resulting from the input of an infinitely narrow pulse of a unit area, termed an impulse. Thus, Fourier transformation of the impulse response generates input impedance, and inverse Fourier transformation of the impedance generates the impulse response^12^,^13^. Although the impulse response of the arterial system is a time−domain equivalent to its input impedance in the frequency domain, they emphasize different aspects of the system.

Regarding the study of the vascular impulse response, the input is an impulse of flow, and the output is the resulting pressure measured in the ascending aorta. In 1980, Sipkema *et al*.^10^ directly applied a flow impulse to the vasculature to study the impulse response of the arterial system. They discovered that the impulse response exhibited an initial sharp peak, followed by an exponential decay with two peaks superimposed on it. An alternative indirect method they employed to obtain the aortic impulse response involved using an inverse Fourier transformation of *Z*_*i*_. This was accomplished through the inverse transformation of *Z*_*i*_ after being filtered by a Dolph–Chebychev weighting function^12^. The Dolph–Chebyshev filter is used to reduce spurious oscillations introduced by truncation of the impedance. Their results demonstrated strong agreement between the directly measured and indirectly derived impulse response functions of the arterial system.

For this study, the impulse response of the arterial system was calculated using an inverse Fourier transform of *Z*_*i*_ by applying a method proposed by Laxminarayan *et al*.^12^. Figure 2 presents the aortic impulse response functions of a healthy rat derived from the measured pressure and *Q*^m^ (2 A), and those calculated from the measured pressure and assumed *Q*^tri^ (2B). The green curve displays the two discrete reflection peaks in the vascular impulse response, which represent pressure reflections linked to the effective reflection sites in the asymmetric T-tube model of the arterial tree^14^. There is no doubt that geometric and elastic tapering and branching of the mammalian arterial tree contribute to multiple reflection sites^3^,^4^. These multiple reflection sites result in diffuse reflections, which characterize the prolonged pressure decay^10^. The presence of two discrete reflection peaks in the vascular impulse response represents pressure reflections from two effective reflection sites^14^. These discrete reflections contribute to the oscillations of moduli and phase in the vascular impedance spectra. It is important to realize that there is no single anatomic structure or change located at these effective reflection sites, but rather they represent the summation of effects of multiple distance reflections. The effective upper body distal to the origin of the subclavian and brachiocephalic vessels generates the first reflection peak and the lower body distal to the descending aorta generates the second reflection peak^10^. Present evidence suggests that these larger vascular reflections probably originate at terminal arterioles^15^. Because the apparent time of the first peak is heavily influenced by early diffuse reflections^15^, we considered the second peak (long arrow in Fig. 2A,B) as the standard for the calculation of *τ*_*w*_ in the lower body circulation. The time of the initial peak (short arrow in Fig. 2A,B) was used as a reference.

According to the Moens and Korteweg formula^16^, as arterial stiffness increases, pulse wave velocity (*c*_*0*_) increases and thereby shortens the traveling time of the forward and reflected pressure waves. Being relatively independent to body shape^3^,^4^,^11^, arterial *τ*_*w*_, which is inversely related to *c*_*0*_, was derived to represent the distensibility of aortas in the lower body circulation; the stiffer the aortic wall is, the shorter the arterial *τ*_*w*_, and vice versa. The CKD rats and both diabetic groups exhibited a decline in aortic distensibility compared with the NC, as manifested by a reduction in *τ*_*w*_^mQ^ (Fig. 5C). An increase in aortic stiffness could also be reflected in the reduction in *τ*_*w*_^triQ^ (Fig. 5F). A reduction in *τ*_*w*_ suggested that CKD and DM caused an early return of pulse wave reflection from the peripheral circulation. Both the CKD and DM contributed to a significant rise in *RM*^mQ^ (Fig. 5A) and *RI*^mQ^ (Fig. 5B), which indicates heavy reflection intensity in the vasculature. Augmented *RM*^triQ^ (Fig. 5D) and *RI*^triQ^ (Fig. 5E) were also observed in the CKD rats and both diabetic groups. Thus, changes in *τ*_*w*_, *RM*, and *RI*, evaluated using either *Q*^m^ or *Q*^tri^ suggest that CKD and DM may modify the timing and magnitude of pulse wave reflection, resulting in an increase in the systolic workload imposed on the heart. These findings indicate that the measured aortic pressure alone, associated with an uncalibrated *Q*^tri^, can reveal arterial wave properties in various diseased animals.

This study has several limitations^17^. Because *Z*_*i*_ cannot be measured in conscious animals, evaluating the effects of pentobarbital anesthesia on rats is impossible. The results reported here pertain only to the measurements made in anesthetized rats in an open-chest condition. This condition might have induced changes in the aortic pressure profiles and introduced reflex effects not found in ordinary conditions. The degree to which anesthesia and thoracotomy influence the pulsatile hemodynamics in rats is uncertain. However, studies conducted using other animals suggest that the effects are minor relative to the biological and experimental variability between animals^18^.

## Conclusions

We propose a method for determining the arterial *τ*_*w*_ from the impulse response of *Z*_*i*_ on the basis of the measured aortic pressure and an assumed triangular flow. The base of the uncalibrated *Q*^tri^ was constructed with a duration set equal to the ejection time derived from measured pressure. After ejection commenced, the timing of the peak triangle was set at the first zero crossing (from above to below) on the fourth-order derivative of the aortic pressure wave. The findings indicate that in addition to wave intensity measurements, *τ*_*w*_ could be approximately calculated using the assumed *Q*^tri^, and exhibited strong correlation with that derived from *Q*^m^. The attractiveness of the study is that flow wave with triangular shape is derived from the measured pressure and that calibration of flow is not essential in the analysis. We suggest that aortic impulse response analysis can be used as an effective method for the estimation of arterial *τ*_*w*_ in the lower body circulation by using a single pressure pulse recording.

## Methods

### Animals and catheterization

Two-month-old male Wistar rats were randomly divided into four groups: (1) NC (*n* = 27), (2) CKD (*n* = 22), (3) type 1 DM (*n* = 22), and (4) type 2 DM (*n* = 11). Under anesthesia with sodium pentobarbital (50 mg kg^−1^; i.p.), CKD was induced by 5/6 subtotal nephrectomy (i.e., right nephrectomy and ligation of two branches of the left renal artery), according to the method by Floege *et al*.^19^. The levels of SCr and BUN were measured with an autoanalyzer (Model 7070, Hitachi Electronics Co., Ltd., Tokyo, Japan). Type 1 DM was induced using a single tail vein injection with 55 mg kg^−1^ of STZ (Sigma, St. Louis, MO, USA) in 0.1 M citrate buffer (pH 4.5) (Sigma, St. Louis, MO, USA)^17^. Type 2 DM was induced by administering intraperitoneally 180 mg kg^−1^ of NA (Sigma, St. Louis, MO, USA) 30 min before an intravenous injection of 50 mg kg^−1^ of STZ dissolved in 0.1 M citrate buffer (pH 4.5)^20^,^21^. The blood glucose level was determined using a SURESTEP Test Strip (Lifescan Inc., Milpitas, CA, USA) to confirm the development of hyperglycemia. Studies on changes in arterial mechanics were performed 8 weeks after the induction of CKD and DM. All rats were allowed free access to Purina chow and water and housed under 12-hour light/dark cycles. The experiments were conducted according to the *Guide for the Care and Use of Laboratory Animals*, and our study protocol was approved by the Animal Care and Use Committee of National Taiwan University.

The general surgical procedures and method used to measure the cardiovascular variables in the anesthetized rats were performed as described previously^17^. In brief, the rats were anesthetized using intraperitoneal sodium pentobarbital (50 mg kg^−1^), placed on a heating pad, intubated, and ventilated with a rodent respirator (Model 131, New England Medical Instruments, Medway, MA, USA). The chest was opened through the second intercostal space on the right side. An electromagnetic flow probe (100 series, internal circumference 8 mm, Carolina Medical Electronics, King, NC, USA) was positioned around the ascending aorta to measure pulsatile *Q*^m^. A high-fidelity pressure catheter (Model SPC 320, size 2 F, Millar Instruments, Houston, TX, USA) was used to measure pulsatile aortic pressure through the isolated carotid artery on the right side. An electrocardiogram (ECG) of lead II was recorded using a Gould ECG/Biotach amplifier (Cleveland, OH, USA). Selective aortic pressure and flow signals from 5–10 beats were averaged in the time domain by using the peak R-wave of the ECG as a fiducial point. The timing asynchronicity between the pressure and flow signals caused by the spatial distance between the flow probe and the proximal aortic pressure transducer was corrected using a time-domain approach, wherein the foot of the pressure waveform was realigned with that of the flow^22^. The resulting aortic pressure and flow signals were subjected to further vascular impedance analysis^4^,^11^,^17^.

### Construction of the unknown flow wave with a triangular shape

The base of the unknown *Q*^tri^ was constructed with a duration set equal to the ejection time derived from measured pressure^8^. The start and end time points of ejection were identified as the intersection of two tangential lines around the foot of the pressure wave (first vertical blue line in Fig. 1) and around the incisura (third vertical blue line in Fig. 1), respectively^17^. The timing of the peak triangle was derived from the fourth-order derivative of pressure waveform (pink curve in Fig. 1). After ejection commenced, the first zero crossing from above to below (second vertical blue line in Fig. 1) determined the peak triangle, which was close to peak of *Q*^m ^8,23. Thus, the unknown flow wave was approximated by a triangular shape, represented by the green curve (*Q*^tri^).

### Impulse response function curve

*Z*_*i*_ was obtained from the ratio of ascending aortic pressure harmonics to the corresponding flow harmonics by using a standard Fourier series expansion technique^4^,^11^,^17^. *Z*_*c*_ was computed by averaging the high-frequency moduli of *Z*_*i*_ data points (fourth to tenth harmonics). The arterial *τ*_*w*_ was computed using the impulse response function of *Z*_*i*_^10^,^15^. This calculation was performed using an inverse Fourier transformation of *Z*_*i*_ after multiplying the first 12 harmonics by a Dolph–Chebychev weighting function with order 24^12^,^17^. The green lines in Fig. 2A,B indicate that the *Z*_*c*_ component of the vascular impulse response was removed. One-half of the time difference between the appearance of the second reflected peak (long arrow) and the initial peak (short arrow) in the impulse response function curve approximates the *τ*_*w*_ in the lower body circulation^10^,^12^.

### Arterial wave separation analysis

Regarding the time domain, the following equations are used to calculate the forward pressure wave (*P*_*f*_) and backward pressure wave (*P*_*b*_):^17^,^24^


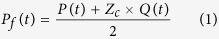



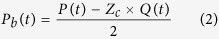


*P*(*t*) is the measured pressure wave, and *Q*(*t*) is either *Q*^m^ or the assumed *Q*^tri^. As mentioned, *Z*_*i*_ is the ratio of pressure and flow quantities. Thus, the product *Z*_*c*_ × *Q*(*t*) in the preceding equations remains unaltered because the calculated *Z*_*c*_ is twice as small when *Q*(*t*) is twice as large. This indicates that an absolute flow calibration is not required. With the measured aortic pressure, the forward and backward pressure waves calculated using *Q*^m^ are illustrated in Fig. 2C, and those calculated using *Q*^tri^ are shown in Fig. 2D. The arterial *RM* was then calculated as the ratio of |*P*_*b*_| and |*P*_*f*_|, i.e., *RM* = |*P*_*b*_|/|*P*_*f*_|^7^. The reflection index (*RI*) is defined as *RI* = |*P*_*b*_|/(|*P*_*f*_| + |*P*_*b*_|).

### Statistics

Results are expressed as the mean ± standard error (s.e.). An independent Student’s *t* test was performed to determine the statistical significance of the results for comparing the effects of CKD and both DM types on arterial wave properties with the NC. Statistical significance was assumed at the level of *P* < 0.05.

## Additional Information

**How to cite this article:** Chang, R.-W. *et al*. Determining arterial wave transit time from a single aortic pressure pulse in rats: vascular impulse response analysis. *Sci. Rep.* **7**, 40998; doi: 10.1038/srep40998 (2017).

**Publisher's note:** Springer Nature remains neutral with regard to jurisdictional claims in published maps and institutional affiliations.

## Figures and Tables

**Table 1:** Baseline characteristics in healthy rats, rats with chronic kidney disease, and rats with either type 1 or type 2 diabetes.

Group	NC (*n* = 27)	CKD (*n* = 22)	type 1 DM (*n* = 22)	type 2 DM (*n* = 11)
BW (g)	455.0 ± 8.2	418.6 ± 8.9	325.9 ± 5.2^†^	408.2 ± 7.8
BS (mg dl^−1^)	96.3 ± 2.0	na	466.6 ± 11.9^†^	157.2 ± 3.1^†^
BUN (mg dl^−1^)	20.07 ± 0.77	65.35 ± 3.10^†^	na	na
SCr (mg dl^−1^)	0.68 ± 0.02	1.68 ± 0.09^†^	na	na
*HR* (beats min^−1^)	395.6 ± 4.9	386.2 ± 7.9	358.2 ± 6.9^†^	365.8 ± 10.7^†^
*P*_*s*_(mmHg)	115.7 ± 2.0	178.1 ± 3.8^†^	121.3 ± 3.5	119.2 ± 4.6
*P*_*d*_ (mmHg)	93.2 ± 2.0	131.8 ± 2.7^†^	96.2 ± 3.3	96.3 ± 4.2
*P*_*m*_ (mmHg)	105.2 ± 2.0	153.9 ± 3.0^†^	109.7 ± 3.4	109.1 ± 4.3
*CO* (ml s^−1^)	2.145 ± 0.081	2.084 ± 0.113	2.347 ± 0.104	1.685 ± 0.109^†^
*R*_*p*_ (mmHg s ml^−1^)	50.9 ± 2.2	77.7 ± 4.0^†^	49.21 ± 3.4	67.1 ± 4.6^†^

All values are expressed as the mean ± s.e. BW, body weight; BS, blood sugar; BUN, blood urea nitrogen; SCr, serum creatinine; *HR*, basal heart rate; *P*_*s*_, systolic aortic pressure; *P*_*d*_, diastolic aortic pressure; *P*_*m*_, mean aortic pressure; *CO*, cardiac output; *R*_*p*_, total peripheral resistance; NC, normal controls; CKD, rats with chronic kidney disease; type 1 DM, streptozotocin-induced diabetic rats; type 2 DM, streptozotocin–nicotinamide-induced diabetic rats; na, not applicable.

*P < 0.05 compared with controls. ^†^*P* < 0.01 compared with controls.

## References

[b1] Blacher J., Asmar R., Djane S., London G. M. & Safar M. E. Aortic pulse wave velocity as a marker of cardiovascular risk in hypertensive patients. Hypertension 33, 1111–1117 (1999). 10334796 10.1161/01.hyp.33.5.1111

[b2] Guerin A. P. . Impact of aortic stiffness attenuation on survival of patients in end-stage renal failure. Circulation 103, 987–922 (2001). 11181474 10.1161/01.cir.103.7.987

[b3] McDonald D. A. Blood flow in arteries (2nd Edition), (Edward Arnold, London, 1974).

[b4] Milnor W. R. Hemodynamics (2nd Edition), (Williams & Wilkins, Baltimore, 1989).

[b5] O’Rourke M. F., Yaginuma T. & Avolio A. P. Physiological and pathophysiological implications of ventricular/vascular coupling. Ann. Biomed. Eng. 12, 119–134 (1984). 6439084 10.1007/BF02584226

[b6] O’Rourke M. F. Arterial stiffness, systolic blood pressure, and logical treatment of arterial hypertension. Hypertension 15, 339–347 (1990). 2180816 10.1161/01.hyp.15.4.339

[b7] Westerhof N., Sipkema P., van den Bos G. C. & Elzinga G. Forward and backward waves in the arterial system. Cardiovasc. Res. 6, 648–656 (1972). 4656472 10.1093/cvr/6.6.648

[b8] Westerhof B. E., Guelen I., Westerhof N., Karemaker J. M. & Avolio A. Quantification of wave reflection in the human aorta from pressure alone: A proof of principle. Hypertension 48, 595–601 (2006). 16940207 10.1161/01.HYP.0000238330.08894.17

[b9] Qasem A. & Avolio A. Determination of aortic pulse wave velocity from waveform decomposition of the central aortic pressure pulse. Hypertension 51, 188–195 (2008). 18172062 10.1161/HYPERTENSIONAHA.107.092676

[b10] Sipkema P., Westerhof N. & Randall O. S. The arterial system characterized in the time domain. Cardiovasc. Res. 14, 270–279 (1980). 7388858 10.1093/cvr/14.5.270

[b11] Nichols W. W. & O’Rourke M. F. McDonald’s blood flow in arteries (6th Edition), (Edward Arnold, London, 2011).

[b12] Laxminarayan S., Sipkema P. & Westerhof N. Characterization of the arterial system in the time domain. IEEE Trans. Biomed. Eng. 25, 177–184 (1978). 640704 10.1109/TBME.1978.326244

[b13] Oppenheim A. V. & Schafer R. W. Discrete-time signal processing (3rd Edition), (Prentice-Hall, New Jersey, 2009).

[b14] O’Rourke M. F. Pressure and flow waves in systemic arteries and the anatomical design of the arterial system. J. Appl. Physiol. 23, 139–149 (1967). 5340142 10.1152/jappl.1967.23.2.139

[b15] Latson T. W., Yin F. C. P. & Hunter W. C. The effects of finite wave velocity and discrete reflection on ventricular loading, in Ventricular/Vascular Coupling: Clinical, Physiological, and Engineering Aspects, (ed. Yin F. C. P.) Ch. 15, 354–383 (Springer-Verlag, New York, 1987).

[b16] O’Rourke M. F. Principles and definitions of arterial stiffness, wave reflections and pulse pressure amplification, in Arterial Stiffness in Hypertension: Handbook of Hypertension series (1st Edition, Vol 23), (ed. Safar M. E. & O’Rourke M. F.) Ch. 1, 3–20 (Elsevier, Amsterdam 2006).

[b17] Chang R. W. . Systolic aortic pressure-time area is a useful index describing arterial wave properties in rats with diabetes. Sci. Rep. 5, 17293, doi: 10.1038/srep17293 (2015). 26620634 PMC4664900

[b18] Cox R. H. Three-dimensional mechanics of arterial segments *in vitro* methods. J. Appl. Physiol. 36, 381–384 (1974). 4814310 10.1152/jappl.1974.36.3.381

[b19] Floege J. . Glomerular cell proliferation and PDGF expression precede glomerulosclerosis in the remnant kidney model. Kidney Int. 41, 297–309 (1992). 1313122 10.1038/ki.1992.42

[b20] Masiello P. . Experimental NIDDM: development of a new model in adult rats administered streptozotocin and nicotinamide. Diabetes 47, 224–229 (1998). 9519717 10.2337/diab.47.2.224

[b21] Chang K. C. . Arterial stiffening and cardiac hypertrophy in a new rat model of type 2 diabetes. Eur. J. Clin. Invest. 36, 1–7 (2006). 10.1111/j.1365-2362.2006.01588.x 16403003

[b22] Mitchell G. F., Pfeffer M. A., Westerhof N. & Pfeffer J. M. Measurement of aortic input impedance in rats. Am. J. Physiol. 267, H1907–H1915 (1994). 7977821 10.1152/ajpheart.1994.267.5.H1907

[b23] Kelly R., Hayward C., Avolio A. & O’Rourke M. F. Noninvasive determination of age-related changes in the human arterial pulse. Circulation 80, 1652–1659 (1989). 2598428 10.1161/01.cir.80.6.1652

[b24] Murgo J. P., Westerhof N., Giolma J. P. & Altobelli S. A. Manipulation of ascending aortic pressure and flow with the Valsalva maneuver: relationship to input impedance. Circulation 63, 122–132 (1981). 7438386 10.1161/01.cir.63.1.122

